# Variability in anthracycline dose conversions and cardiotoxicity monitoring: insights from hospital pharmacists on institutional protocols in oncology practice

**DOI:** 10.1007/s00520-026-10774-z

**Published:** 2026-05-14

**Authors:** Sheraz A. Ditta, Franny Jongbloed, Olivier C. Manintveld, Jan-Dietert Brugma, Nick Wlazlo, Monique E. M. M. Bos, Hugo M. van der Kuy, Ron H. J. Mathijssen

**Affiliations:** 1 Department of Medical Oncology, Erasmus MC Cancer Institute, Rotterdam, the Netherlands; 2 Department of Medical Oncology, Leiden University Medical Center, Leiden, the Netherlands; 3 Department of Cardiology, Erasmus University Medical Center, Rotterdam, the Netherlands; 4 Department of Outpatient Pharmacy, Erasmus University Medical Center, Rotterdam, the Netherlands; 5 Department of Hematology, Erasmus University Medical Center, Rotterdam, the Netherlands; 6 Department of Medical Oncology, Maastricht University Medical Center, Maastricht, the Netherlands; 7 Department of Hospital Pharmacy, Erasmus University Medical Center, Rotterdam, the Netherlands

**Keywords:** Doxorubicin, Anthracycline, Cardiotoxicity, Cumulative dose, Equivalence factor, Chemotherapy

## Abstract

**Purpose:**

Anthracycline-induced cardiotoxicity is dose-dependent, yet guidelines provide inconsistent cumulative dose thresholds and few standards for converting doses when multiple agents are used. Moreover, no formal guideline exists to unify these practices and responsibilities. Therefore, we evaluated anthracycline dosing and cardiotoxicity monitoring across Dutch hospitals to identify variability.

**Methods:**

A national cross-sectional survey was conducted among hospital pharmacists in the first quarter of 2024 in the Netherlands. Hospitals completed a 16‑item questionnaire assessing cumulative dose thresholds, equivalence factors for converting to doxorubicin‑equivalent doses, cardiac monitoring protocols and circumstances under which thresholds are exceeded.

**Results:**

Responses were received from 43 of 69 hospitals (62%). Reported cumulative dose thresholds varied widely for idarubicin (150—450 mg/m^2^) and mitoxantrone (80—250 mg/m^2^); equivalence factors differed more than ten‑fold between institutions. Nearly half of respondents reported exceeding recommended cumulative dose thresholds in specific clinical contexts. Cardiac monitoring was inconsistent, with 65% of hospitals not routinely measuring left ventricular ejection fraction. Moreover, multigated acquisition scans were used more often than echocardiography despite guideline preference for the latter.

**Conclusion:**

Our results reveal substantial heterogeneity in anthracycline dosing and cardiotoxicity monitoring across hospitals. Variability in equivalence factors, particularly for mitoxantrone and idarubicin, may lead to misestimation of cumulative cardiotoxic risk. These findings support the need for standardized approaches to dose conversion and improved adherence to cardiac monitoring recommendations to enhance patient safety.

**Supplementary Information:**

The online version contains supplementary material available at 10.1007/s00520-026-10774-z.

## Introduction

Anthracyclines are potent chemotherapeutic agents and a cornerstone in treating various malignancies, including haematological cancers, sarcomas, and solid tumours [1]. However, their clinical use is limited by dose-dependent cardiotoxicity [2]. International guidelines provide recommendations on maximum cumulative dose thresholds for individual anthracyclines, but these provide limited guidance on how to calculate total cumulative exposure across different agents. As a result, clinical interpretation varies, and no formal national strategy exists in the Netherlands to unify these practices [3, 4].

First introduced in the 1960 s, anthracyclines such as doxorubicin, daunorubicin, and epirubicin exert their antitumour effects through DNA intercalation, inhibition of topoisomerase II, and the generation of reactive oxygen species (ROS) [5]. While effective, these mechanisms contribute to off-target toxicities [6]. Cardiac tissue, particularly cardiomyocytes, is highly susceptible to oxidative damage due to relatively low levels of endogenous antioxidant enzymes [7]. Anthracyclines exacerbate this vulnerability by impairing mitochondrial function, increasing ROS production, and reducing ATP synthesis, ultimately triggering apoptosis [8, 9]. Anthracycline-induced cardiotoxicity (AIC) can manifest as acute (within hours to weeks), early-onset chronic (within the first year), late-onset chronic (emerging years to decades later), or subclinical forms detectable only through imaging or biomarkers [10, 11]. The estimated incidence ranges from < 1% for acute, 1–5% for early-onset, 5–20% for late-onset, and up to 30–50% for subclinical cardiotoxicity, depending on cumulative dose, patient characteristics, and monitoring methods[2, 12–14]. The incidence of cardiotoxicity varies among different anthracyclines, with each agent having specific dose-dependent risks. For example, doxorubicin has been associated with an incidence of 2.2% in an early study and 4.6% for cardiotoxicity in a recent retrospective cohort study of breast cancer patients[15, 16]. Similarly, epirubicin showed an incidence of 3.7 per 1000 person-years for symptomatic heart failure in long-term follow-up, with short-term heart failure incidence exceeding 5–10% at high doses (~ 900 mg/m^2^) [17, 18]. Daunorubicin has shown an incidence of 10.0% for congestive heart failure in a very early randomized trial comparing vincristine/prednisone versus vincristine/prednisone plus daunorubicin, and a more recent study showed a 7.1% cumulative incidence of cardiotoxicity at 2 years of treatment in patients post stem cell transplant [19, 20]. Idarubicin is associated with a 5–11% incidence of cardiomyopathy at cumulative doses of 150–290 mg/m^2^, with 18% showing mild asymptomatic LVEF decreases, and mitoxantrone has been linked to 14% de novo cardiotoxicity in patients with leukaemia and multiple sclerosis at a mean cumulative dose of 59.7 mg/m^2^ [21, 22]. These examples highlight the variability in cardiotoxicity risk associated with different anthracyclines and reinforce the need for careful monitoring in patients receiving these agents.

Doxorubicin, discovered in the late 1960 s as a hydroxylated analogue of daunorubicin, became the standard drug due to its broad efficacy in solid tumours and hematologic malignancies [23]. In contrast, daunorubicin is primarily used for hematologic cancers. Epirubicin was developed to reduce cardiotoxicity; however, it is less widely used than doxorubicin and in higher doses impacting LVEF comparable to doxorubicin [23]. Idarubicin, a lipophilic derivative of daunorubicin, is mainly used for acute myeloid leukaemia (AML), especially in adults due to its higher cardiotoxicity risk [23]. Lastly, mitoxantrone, designed to be less cardiotoxic, still carries a relevant risk of cardiomyopathy and is therefore limited to treating certain leukaemia’s and multiple sclerosis [23]. Despite the development of these analogues, doxorubicin remains the benchmark anthracycline due to its extensive clinical history, broad efficacy, and proven survival benefits, making it the standard of care for many cancers [23]. In pediatric oncology, daunorubicin is commonly used for acute lymphoblastic leukaemia, while doxorubicin is preferred for solid tumours [24–26]. Idarubicin is also used for acute myeloid leukaemia in both pediatric and adult patients [23–25]. In adult oncology, doxorubicin remains a first-line treatment for various cancers, including breast cancer and lymphomas, while epirubicin is preferred/prescribed in some cases due to its lower cardiotoxicity risk [24, 25]. Since the risk of AIC is strongly dose-dependent, careful monitoring of cumulative exposure remains essential [2].

Multiple formulations and analogues of anthracyclines exist, each with distinct toxicity profiles and clinical applications [1]. Pegylated liposomal doxorubicin is often considered less cardiotoxic than conventional doxorubicin and is sometimes preferred for patients with underlying cardiac risks [27, 28]. Nevertheless, cumulative dosing must still be considered when patients receive sequential or combination regimens involving anthracyclines. In case of these sequential or combination regimens, clinicians rely on dose equivalence factors to convert administered doses into doxorubicin-equivalent doses to estimate cumulative cardiotoxic risk [26]. However, these equivalence factors are not standardized and may differ substantially. An overview of often cited maximum cumulative thresholds for each anthracycline is shown in Table 1, and an overview of often cited dose conversion factors is shown in Table 2. Despite the clinical importance of accurate dose conversions, it remains unknown how anthracyclines are dosed and monitored in daily practice.

**Table 1:** Overview of cumulative anthracycline dose thresholds from international and national sources

Anthracyclines and analogues	ESMO guidelines [29]	PW [34]	SmPC [44–49]^a^	UK NHS institutional protocols [30–33]^b^
Doxorubicin (mg/m^2^)	500	400–550	550	450–550
Doxorubicin liposomal (mg/m^2^)	900	NA	450	450–550
Daunorubicin (mg/m^2^)	NA	400–550	500–600	600
Epirubicin (mg/m^2^)	720	720–900	900	900–1000
Idarubicin (mg/m^2^)	90	150–290	150–290	150
Mitoxantrone (mg/m^2^)	120	80–120	72–140	140–160

^a^According to manufacturer recommendations

^b^Values derived from UK regional chemotherapy protocols (SWAG Cancer Alliance); not part of formal national or ESC/ESMO guidelines

Abbreviations: *ESMO* European Society of Medical Oncology, *PW* Pharmaceutisch Weekblad, *SmPC* Summary of Product Characteristics, *UK NHS* United Kingdom National Health Services, *NA* not available

**Table 2:** Reported anthracycline dose equivalence factors across different literature sources

Anthracyclines and analogues	CCSS[50]	COG [51]	ESC [3]	Feijen et al.[26]	Keefe et al.[52]	PW [34]
Doxorubicin (ref)	1	1	1	1	1	1
Daunorubicin	1	0.5	0.6	0.6	0.5	0.5
Epirubicin	NA	0.67	0.8	0.8	0.5	0.5
Idarubicin	3	5	5	NA	2	2
Mitoxantrone	NA	10	10.5	10.5	2.2	2.2

Values represent the equivalence factor used to convert the administered dose of each anthracycline into doxorubicin-equivalent milligrams for cardiotoxicity risk assessment

Abbreviations: *CCSS* Childhood Cancer Survivor Study, *COG* Children’s Oncology Group, *ESC* European Society of Cardiology, *PW* Pharmaceutisch Weekblad, *NA* not applicable

Therefore, this study investigates current anthracycline dosing practices across Dutch hospital pharmacies via a national survey. By identifying variability in cumulative dose thresholds, equivalence factors, and cardiac monitoring approaches, we aim to identify variability and support the need for a standardized practice. Such standardization may help to optimise anthracycline use and reduce cardiotoxicity risk, particularly in high-risk patients.

## Methods

### Study design and data collection

We conducted a cross-sectional survey of hospital pharmacies in the Netherlands between January 2024 and April 2024. Pharmacists were identified through the Dutch Association of Hospital Pharmacies (NVZA). One response per hospital organization was requested to avoid duplication. The survey was distributed digitally and comprised 16 questions (2 closed-ended, 14 open-ended). A reminder was sent out after 4 weeks. The survey covered cumulative dose thresholds, use of equivalence factors, cardiac monitoring practices, and situations where thresholds are exceeded. Respondents were asked to report the cumulative dose thresholds used in their institution, for each individual anthracycline. We did not impose conversion to a fixed equivalence factor; reported thresholds were analysed a as provided by the respondent. Respondents were also asked to specify the conversion factors used in their institution for calculating total cumulative anthracycline exposure. The full survey can be found as Supplementary Material 1.

### Data Management and Analysis

A national survey was conducted using Microsoft Forms (Microsoft Corporation, Redmond, WA). Data were collected anonymously. Open-text responses were grouped thematically for reporting. No statistical comparisons were made. Descriptive analysis was used to summarize survey findings using SPSS 28.0.1.0 software (IBM SPSS, Chicago, IL) and GraphPad Prism Software Version 9.0 (GraphPad Software Inc., San Diego, CA). All survey responses were reviewed and validated by a second researcher.

### Ethical Considerations

The study did not involve patient data or interventions and was therefore exempt from formal ethical approval under Dutch law. Participation was voluntary, and informed consent to use the data from the survey was implied by survey completion. No personal identifiers were collected.

## Results

### Responders

All 69 hospital organizations with hospital pharmacies in the Netherlands were invited to participate in the survey. Of these hospital pharmacies, 43 completed the questionnaire, resulting in a response rate of 62%.

### Cumulative dose thresholds

**Fig. 1:**
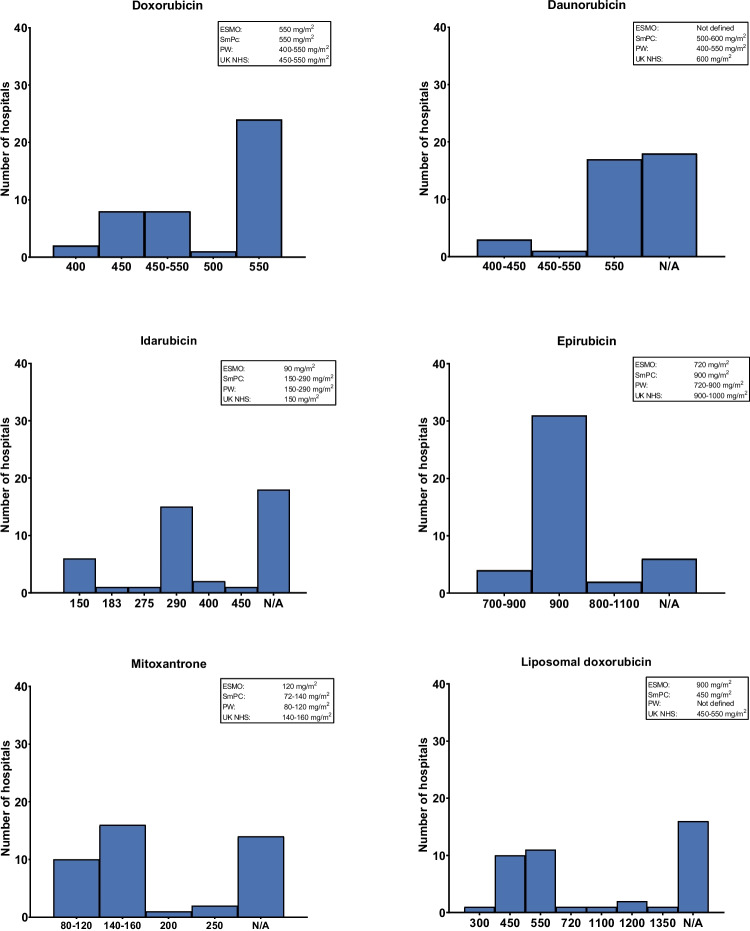
Reported cumulative anthracycline dose thresholds by hospital. Abbreviations: ESMO = European Society of Medical Oncology; PW = Pharmaceutisch Weekblad; UK NHS = United Kingdom National Health Service institutional protocols

Survey results as well as reference thresholds from international and national sources are shown in Fig. 1 for each individual anthracycline. An overview of these reference thresholds is also shown in Table 1. Thresholds for doxorubicin were most frequently set at 550 mg/m^2^ (*n* = 24), although several institutions (*n* = 16) reported using ranges between 450 and 550 mg/m^2^. For daunorubicin, 17 hospitals used a threshold of 550 mg/m^2^, while another 18 reported not to use daunorubicin at all. The remaining hospitals reported a threshold range between 400–550 mg/m^2^. Idarubicin thresholds were highly variable, with the most common value being 290 mg/m^2^ (*n* = 15), but values ranged threefold; between 150 and 450 mg/m^2^. Epirubicin thresholds were more consistently reported, with 31 hospitals adhering to 900 mg/m^2^ and four hospitals used a range of 700–900 mg/m^2^. Notably, four hospitals had set the threshold between 700–900 mg/m^2^ and two hospitals conformed to a higher range of 800–1100 mg/m^2^. Mitoxantrone and pegylated liposomal doxorubicin both had broad variation, with mitoxantrone having reported thresholds from 80 to 250 mg/m^2^ and pegylated liposomal doxorubicin from 300 to 1350 mg/m^2^. A majority of the hospitals prescribing mitoxantrone (*n* = 16) used 140–160 mg/m^2^ as maximum for mitoxantrone, while 10 hospitals used 80–120 mg/m^2^ as a threshold. A minority used a higher range, of 200 mg/m^2^ (*n* = 1) and 250 mg/m^2^ (*n* = 2), respectively. For pegylated liposomal doxorubicin, thresholds varied from 300–1350 mg/m^2^, with the majority using 450 mg/m^2^ (*n* = 10) or 550 mg/m^2^ (*n* = 11), respectively.

### Dose equivalence factors

**Fig. 2:**
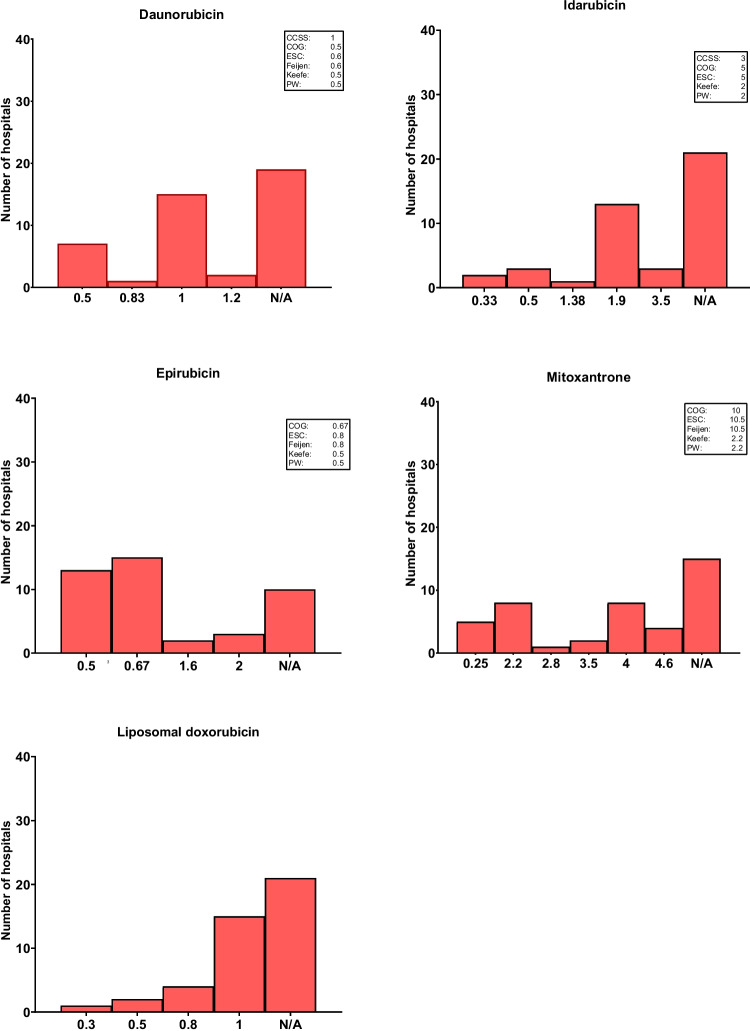
Reported equivalence factors for anthracyclines by hospital. Abbreviations: CCSS = Childhood Cancer Survivor Study; COG = Children’s Oncology Group; ESC = European Society of Cardiology; PW = Pharmaceutisch Weekblad

Survey results for dose equivalence factors, as well as references from international and national sources, are shown in Fig. 2. An overview of these reference equivalence factors (to convert the administered dose of each anthracycline into doxorubicin-equivalent doses) is also shown in Table 2. The reported equivalence factors for daunorubicin showed a wide variation. Seven hospitals used a factor of 0.5, while 15 used a factor of 1, resulting in a 100% difference. The remainder reported values of 0.83 (*n* = 1) or even 1.2 (*n* = 2). Among the 24 hospitals using idarubicin, 13 used a factor of 1.9, while others reported factors between 0.33 and 3.5, resulting in a more than tenfold difference between the extremes. Epirubicin equivalence factors ranged broadly as well. While 27 hospitals used a factor between 0.5 and 0.67, a few used factors between 1.6 and 2 (*n* = 5). For mitoxantrone reported values ranged widely from 0.25 to 4.6, compared to 2.2–10.5 in the referenced guidelines. Pegylated liposomal doxorubicin has reported factors between 0.3 and 1, but no consensus or standard factor was evident in national or international guidelines.

### Cardiac monitoring practices

**Fig. 3:**
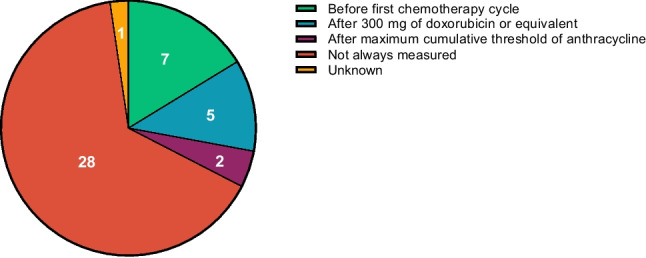
Distribution of cardiac function monitoring practices across Dutch hospitals

**Fig. 4:**
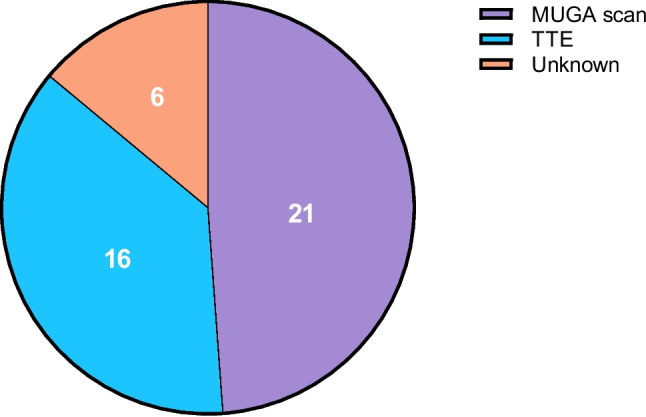
Diagnostic tools used for cardiac function evaluation during anthracycline therapy. Abbreviations: TTE = Trans Thoracic Echocardiography; MUGA = Multigated Acquisition

Most hospitals (*n* = 28, 65%) reported that cardiac function is not routinely measured prior to anthracycline initiation and during follow up. Only seven hospitals (16%) perform a cardiographic assessment before the first chemotherapy cycle and five (12%) after patients have received 300 mg/m^2^ of doxorubicin or its equivalent. Two hospitals (5%) do the assessment after the maximal recommended cumulative dose is reached (Fig. 3**)**. The most commonly used techniques are MUGA (48%) and echocardiography (37%; Fig. 4). Electrocardiograms (14%) and lab tests (12%) were also occasionally performed during follow up. The lab tests were according to the European Society of Cardiology guidelines, which state that cardiac troponins and natriuretic peptides (BNP or NT-proBNP) should be used to detect early and late anthracycline-induced cardiotoxicity [3]. Regarding threshold exceedance, 47% of hospitals reported that patients occasionally receive anthracyclines above the recommended cumulative dose.

## Discussion

This nationwide survey reveals substantial heterogeneity in anthracycline dosing practices among Dutch hospitals. While international guidelines, such as the ESC 2022 cardio-oncology guideline, provide cumulative dose thresholds for individual anthracyclines, these guidelines do not specify standardized equivalence factors for converting between agents [3, 29]. Some European countries, such as the United Kingdom (e.g., SWAG Cancer Alliance protocols), have regionally standardized chemotherapy protocols, but these are not official national guidelines [30–33]. Our findings demonstrate marked variation in how these recommendations are interpreted and applied in clinical practice. This suggests that the current challenge is not the absence of guidance, but more in the lack of standardized (inter)national implementation.

We observed considerable variation in anthracyclines dosing between institutions. Similarly, reported equivalence factors for converting doses to doxorubicin equivalents showed significant inconsistency. Some Dutch institutions reported to use pharmacy-authored resources such as those by Brugma et al. (2010) and de Boer et al. (2006) to guide cumulative dose calculations and conversion factors [34, 35]. While these documents offer practical tools for clinical use, these are not formal guidelines and these also differ in key aspects from more recent evidence. For example, Brugma et al. recommended an equivalence factor of 2.2 for mitoxantrone, whereas Feijen et al., a decade later, reported an equivalence factor exceeding 10 based on cardiotoxicity outcomes in a large population-based cohort [26, 34]. This discrepancy likely reflects the limited direct evidence on mitoxantrone-induced cardiotoxicity, as few studies have systematically evaluated its dose–response relationship. The uncertainty surrounding its true cardiotoxic potential may partly explain the wide variation in equivalence factors used by hospitals. Tables 1 and 2 illustrate these inconsistencies, showing not only variation between hospital-reported values and literature but also divergence among international guidelines themselves. This variability partly reflects the heterogeneous evidence base on which conversion factors are derived. Some originate from early pharmacological or in vitro studies, others from small clinical cohorts, and more recent ratios from large epidemiological studies linking cumulative dose to observed cardiotoxic events [1, 15, 26, 34–36]. Consequently, many published factors rely on limited or outdated data. Such inconsistencies complicate clinical decision-making and increase the risk of over- or underestimating cumulative cardiotoxic burden, particularly in sequential or combination regimens.

Although international societies such as the ESC and the European Society for Medical Oncology (ESMO) provide general recommendations for monitoring AIC, these guidelines are often limited to baseline assessments, with more detailed monitoring strategies left to local discretion [3, 29]. Importantly, both guidelines recommend echocardiography as the preferred imaging modality for assessing cardiac function because of its ability to evaluate diastolic function, global longitudinal strain, and structural abnormalities. MUGA scans also expose patients to ionizing radiation and an intravenous injection/infusion, which is unfavourable for repeated monitoring or for younger patients treated with curative intend [37]. MUGA is only recommended when echocardiography is not feasible or yields poor image quality [3, 29]. In contrast, our findings show that MUGA was used more frequently than echocardiography in Dutch hospitals. One possible explanation is that MUGA scans may be more readily available in certain institutions, with shorter waiting times and greater logistical capacity compared to echocardiography. Another possible reason could be historical practice, as MUGA was long regarded as a standard method for left ventricular ejection fraction (LVEF) monitoring in oncology [38]. Therefore, this practice may leave patients at risk of undetected cardiac dysfunction or unnecessary invasive procedure and radiation. Recent evidence supports that initiating cardio-protective therapy, such as angiotensin-converting enzyme (ACE) inhibitors, during anthracycline treatment, particularly in response to rising cardiac biomarkers, can help preserve LVEF and reduce cardiac injury [39]. Another recent study showed that dexrazoxane also has cardio protective potential, however this was studied ex vivo [40]. This reinforces the importance of imaging and biomarker surveillance strategies beyond baseline assessments, including guidance on the frequency and timing of follow-up imaging after intravenous anthracycline administration [39, 41–43].

Interestingly, nearly half of the hospitals reported exceeding cumulative dose thresholds under specific clinical circumstances, such as leukaemia protocols, palliative regimens such as low-dose weekly dosing schemes (e.g. 15 mg of doxorubicin). In leukaemia cases, particularly with AML, exceeding the threshold was seen in high-risk adult patients where curative intent took precedence. In palliative settings, anthracyclines were sometimes used above the threshold when no better treatment options were available, and the benefit-risk ratio favoured continuation. Additionally, low-dose weekly regimens of doxorubicin were commonly mentioned as situations where the threshold was exceeded under careful monitoring. These decisions were generally made in close collaboration between pharmacists and treating physicians, often based on individualized risk–benefit evaluations. While such personalized care is essential in oncology, the exceedance of recommended thresholds without a uniform framework raises important questions about how risks are assessed and communicated across institutions.

A clear international guideline is particularly valuable to provide a structured reference point, allowing clinicians to consciously and transparently deviate from recommendations when clinical benefit, shared decision-making or expert opinion justifies it. Without such a framework, incorrect dosing conversions or threshold exceedance may result in delayed detection of cardiac dysfunction, especially in patients treated with curative intent or in combination regimens involving agents such as trastuzumab, pertuzumab, or thoracic radiotherapy. In addition, the survey findings align with broader discussions in cardio-oncology about survivorship care, especially for young patients and those also exposed to left-sided chest radiation. In this context, the absence of structured follow up protocols with adequate biomarkers reflects another missed opportunity for prevention.

Some limitations should be acknowledged. First, the survey was completed by hospital pharmacists, who are primarily responsible for tracking cumulative anthracycline dosing. In the Dutch healthcare system, hospital pharmacists are closely integrated within the clinical team and are involved in treatment protocols and dosing decisions. However, responses may still reflect institutional practice rather than all aspects of bedside clinical decision-making. In addition, while respondents could indicate circumstances in which thresholds were exceeded, the full clinical rationale behind these decisions could not be systematically captured. Second, our data were collected at the hospital level, so the finding that 47% of hospitals occasionally exceeded thresholds does not directly translate to patient-level frequency, which may be relatively low and clinically justified. Third, although the response rate was relatively high (62%), results may not fully represent all Dutch institutions, especially as not all hospitals prescribe all anthracyclines; this may partly mitigate bias. Fourth, as with all surveys, responses are subject to reporting bias and possible variation in interpretation across institutions. Finally, the open-ended format of some questions led to incomplete answers and potential categorization bias, although independent verification by a second reviewer partly reduced this risk. Despite these limitations, the survey provides a unique overview of anthracycline dosing practices in a European country with high standards of care and underscores the need for standardized guidance.

In summary, our results highlight substantial heterogeneity in anthracycline dosing and cardiotoxicity monitoring practices across Dutch hospitals. Notably, equivalence factors for different anthracyclines vary significantly, and reliance on outdated local resources can result in the wrong estimation of cumulative cardiotoxic risk. Based on these findings, we recommend standardization of equivalence factors for mitoxantrone and idarubicin, as these agents are associated with higher cardiotoxicity risks compared to what is now frequently believed in clinical practice and are still in use in curative setting. Additionally, we emphasize the need for awareness of guidelines for cardiac monitoring, especially in high-risk patient populations receiving higher total doses of anthracyclines. These measures are crucial for improving patient safety and ensuring the effective use of anthracyclines in clinical practice. Future adequately powered studies are needed to define optimal dose thresholds and equivalence factors, thereby strengthening evidence-based recommendations.

## Supplementary Information

Below is the link to the electronic supplementary material.

ESM 1 (DOCX 18.4 KB)

## Data Availability

The datasets generated and analysed during the current study are not publicly available due to institutional privacy restrictions but are available from the corresponding author on reasonable request.
