# Supplementary material for: Variability in anthracycline dose conversions and cardiotoxicity monitoring: insights from hospital pharmacists on institutional protocols in oncology practice
Source: Support Care Cancer. 2026 May 14;34(6):541. doi: 10.1007/s00520-026-10774-z (PMC13176006; doi:10.1007/s00520-026-10774-z)
Supplement: Supplementary file 1 — (DOCX 18.4 KB) [file 520_2026_10774_MOESM1_ESM.docx]

**Supplementary Material 1. Survey Questionnaire Distributed to the Dutch Hospital Pharmacies***

*Original survey was sent in Dutch

## General questions

1. When does LVEF get measured for anthracycline based chemotherapy?
   1. Before first chemotherapy cycle
   2. After 300 mg/m^2^ doxorubicin (or equivalent dose of other anthracycline)
   3. After max dose has been received
   4. We don't always measure
2. Which technique is used to measure LVEF?
   1. MUGASCAN
   2. Echocardiography
   3. MRI
3. Are there other parameters measured, besides LVEF?
4. Do patients ever receive anthracyclines above the toxicity threshold? If so, when does this happen?

## Anthracycline related questions

1. What is the toxicity threshold (mg/m^2^) of doxorubicin in your hospital?

- 1. What is the toxicity threshold (mg/m^2^) of daunorubicin in your hospital?
  2. Dose conversion factor (in relation to doxorubicin)?
  3. What is the toxicity threshold (mg/m^2^) of epirubicin in your hospital?
  4. Dose conversion factor (in relation to doxorubicin)?
  5. What is the toxicity threshold (mg/m^2^) of mitaxontron in your hospital?
  6. Dose conversion factor (in relation to doxorubicin)?
  7. What is the toxicity threshold (mg/m^2^) of idarubicin in your hospital?
  8. Dose conversion factor (in relation to doxorubicin)?

- 1. What is the toxicity threshold (mg/m^2^) of liposomal doxorubicin in your hospital?
  2. Dose conversion factor (in relation to doxorubicin)?
